# Advances in research on RNA methylation and its role in the immune microenvironment of gastrointestinal tumors

**DOI:** 10.3389/fcell.2026.1774623

**Published:** 2026-06-03

**Authors:** Yushuo Duan, Ziyi Xu, Kaixiang Wen, Chen Xue, Ruile Shen, Xinyu Gu

**Affiliations:** 1 Department of Neurology, The First Affiliated Hospital, College of Clinical Medicine of Henan University of Science and Technology, Luoyang, China; 2 Henan Key Laboratory of Cancer Epigenetics, Cancer Institute, The First Affiliated Hospital, College of Clinical Medicine of Henan University of Science and Technology, Luoyang, China; 3 State Key Laboratory for Diagnosis and Treatment of Infectious Diseases, National Clinical Research Center for Infectious Diseases, National Medical Center for Infectious Diseases, Collaborative Innovation Center for Diagnosis and Treatment of Infectious Diseases, The First Affiliated Hospital, Zhejiang University School of Medicine, Hangzhou, China

**Keywords:** gastrointestinal tumors, immune evasion, immune prognostic model, RNA methylation, tumor immune microenvironment

## Abstract

RNA methylation is a key epigenetic regulatory mechanism that precisely regulates gene expression through the dynamic and reversible actions of “Writers,” “Erasers” and “Readers”. In recent years, RNA methylation has been widely implicated in tumor formation and immune regulation. For instance, in gastrointestinal tumors, the immunosuppressive state of the tumor microenvironment (TME) is an important factor driving cancer progression. Research indicates that RNA methylation restructures the tumor immune microenvironment (TIME) by modulating tumor and immune cell functions and reprogramming immune metabolism. This process thereby regulates immune evasion and treatment response. Therefore, a better understanding of the mechanism of RNA methylation affecting immune regulation in gastrointestinal tumors can not only help elucidate new patterns of tumor development but also provide important evidence for developing new strategies that combine targeted RNA methylation with immunotherapy. This article aims to review the latest research progress in this field and looks forward to potential clinical applications.

## 1 Introduction

Malignant tumors, especially gastrointestinal tumors, continue to be a major public health burden worldwide, with their occurrence, development, and therapy resistance involving complex molecular mechanisms. The primary malignant tumors of the gastrointestinal tract are hepatocellular carcinoma (HCC), colorectal cancer (CRC), gastric cancer (GC), pancreatic ductal adenocarcinoma (PDAC), and esophageal cancer (ESCA) (Arnold et al., 2020). The occurrence and development of gastrointestinal tumors are driven not only by the tumor cells themselves but also mediated by the tumor microenvironment (TME). The TME consists of a variety of cell types, such as immune cells, cancer-associated fibroblasts (CAFs) and endothelial cells (Ye et al., 2022; De Visser and Joyce, 2023). It is increasingly recognized that the functional core of the TME comprises its immune components, also known as the tumor immune microenvironment (TIME).

The TIME is a complex ecosystem consisting of tumor cells, immune cells, immunosuppressive molecules, and chemokines (Lv et al., 2022). This microenvironment plays a crucial role in tumor progression, invasion, metastasis, immune evasion, and treatment resistance (de Visser and Joyce, 2023; Lv et al., 2022). In gastrointestinal tumors, the TIME often shows an immunosuppressive state, which is primarily characterized by immune cell dysfunction, including the exhaustion of effector cells like T cells and natural killer (NK) cells, as well as the unusual growth of regulatory T cells (Tregs), myeloid-derived suppressor cells (MDSCs), and M2-type tumor-associated macrophages (TAMs). This immunosuppressive state is also accompanied by a high concentration of immune checkpoint molecules, suppressive cytokines, and certain chemokines. Furthermore, metabolic alterations in tumor cells and immune cells can also indirectly shape this immunosuppressive environment (Kim, 2018). The immunosuppressive nature of the TIME is a vital factor in why tumors evade immune surveillance, leading to resistance to immunotherapy. Therefore, a thorough understanding of what makes up the TIME and how it works is key to developing new immunotherapies and overcoming treatment resistance.

Over the past decade, RNA epitranscriptomics has emerged as a new Frontier in epigenetics, offering key insights into tumor biology (Huang et al., 2025). RNA methylation as its core branch includes several modifications such as N6-methyladenosine (m6A), 5-methylcytosine (m5C), N1-methyladenosine (m1A), and N7-methylguanosine (m7G). These modifications regulate RNA metabolism and function via “Writers,” “Erasers” and “Readers,” which are widely involved in processes such as tumorigenesis, immune system evasion, and drug resistance (Cheng, 2025). Furthermore, the role of the immune system in tumor progression is becoming increasingly evident. Research indicates that RNA methylation plays a key role in the progression of gastrointestinal tumors by simultaneously regulating tumor and immune cell functions: it promotes tumor growth by reshaping the TIME, thereby becoming a crucial link between genetic information and immune traits (Yang et al., 2023).

Based on the above, a comprehensive understanding of the RNA epitranscriptional regulatory processes during the progression of gastrointestinal tumors is essential for fully understanding how tumor cells interact with the TIME. This review aims to provide a systematic overview of RNA methylation focusing on gastrointestinal tumors, and highlight its crucial role in shaping and functioning within the TIME, with the hope of uncovering new mechanisms of tumor immune evasion and to offer a theoretical foundation and potential new targets for developing combination immunotherapy strategies based on RNA modifications.

## 2 Overview of RNA methylation

RNA methylation, as a core mechanism of epitranscriptomics, precisely regulates RNA splicing, export, stability, translation, and degradation (Table 1), thereby widely participating in the regulation of key pathological processes such as oncogenesis, metabolic disorders, and immune responses (Cheng, 2025).

**TABLE 1:** Types of RNA methylation and their regulatory mechanisms.

Methylation	Regulator		Function	References
m6A	Writer	METTL3-METTL14	Core catalytic unit of MTC.	Dou et al. (2023)
		METTL16	Targets U6 snRNA and selective pre-mRNAs/ncRNAs	Warda et al. (2017)
		ZCCHC4	Modifies 28S rRNA to regulate protein synthesis	Pinto et al. (2020)
	Eraser	FTO	Modulates energy metabolism and tumorigenesis via mRNA regulation	Shen et al. (2022), You et al. (2022)
		ALKBH5	Regulates mRNA splicing, nuclear export, stability, and translation	Shen et al. (2022)
	Reader	YTHDF1/2/3,YTHDC1/2	Regulates mRNA translation, degradation, splicing, and nuclear export	Deng et al. (2023)
		hnRNP C, hnRNP G	Regulates pre-mRNA splicing	Li et al. (2024a)
		HNRNPA2B1	Mediates primary miRNA processing	Li et al. (2024a)
		IGF2BP1/2/3	Enhances mRNA stability and promotes its translation	Deng et al. (2023)
m5C	Writer	NSUN1-7	NSUN1/5 target 28S rRNA; NSUN2 modifies diverse RNAs; NSUN3 methylates mt-tRNA; NSUN4 modifies small subunit rRNA.	Yang et al. (2023), Li et al. (2022a)
		DNMT2	Maintains tRNA stability and regulates translation of specific genes; Modulates mRNA methylation	Lu et al. (2024) Li et al. (2022a)
		METTL17	Regulates the translation of mt-RNA.	Li et al. (2024b)
	Eraser	TET1, TET2	Generates and accumulates hm5C from m5C oxidation	Fu et al. (2014), He et al. (2011)
		ALKBH1	Converts hm5C into f5C through oxidative modification	Arguello et al. (2022)
	Reader	ALYREF	Regulates mRNA nuclear export by recognizing specific sequence motifs	Fan et al. (2019)
		YBX1	Functions in RNA metabolism and DNA repair	Wang et al. (2025a)
		FMRP	Recognizes specific target mRNAs to regulate their translation in neurons	Zhao et al. (2025a)
m1A	Writer	TRMT6-TRMT61A	Catalyzes m1A modification at position 58 of tRNA to maintain its structural stability	Pajdzik et al. (2024), Liu et al. (2022a)
		TRMT61 B-TRMT10C	Maintains the stability and function of mt-tRNA.	Smoczynski et al. (2024)
		RRP8	Catalyzes 28S rRNA to ensure proper maturation of functional ribosomes	Zhu et al. (2018)
	Eraser	ALKBH3	Modulates RNA biology via tRNA and mRNA modification	Yang et al. (2023), Li et al. (2016)
		ALKBH1	Governs translation efficiency through regulation of mature tRNA production	Liu et al. (2016)
	Reader	YTHDF1/2/3, YTHDC1	Binds m1A	Dai et al. (2023), Dai et al. (2018)
m7G	Writer	RNMT-RAM	Catalyzes the synthesis of the m7G cap at the 5′end of mRNA within the nucleus	Bueren-Calabuig et al. (2019)
		METTL1-WDR4	Catalyzes internal m7G modifications within tRNAs, miRNAs, and mRNAs	Ruiz-Arroyo et al. (2023)
		WBSCR22-TRMT112	Catalyzes rRNA m7G modification to ensure ribosome maturation and function	Zhang et al. (2023a)
	Reader	eIF4E	Recognizes the m7G cap at the 5′end of mRNA to initiate translation	Sato and Maquat (2009)
		CBC	Mediates mRNA processing within the nucleus and its export to the cytoplasm	Mars et al. (2021)
		QKI5/6/7	Regulates mRNA stability and translation efficiency under stress conditions	Han et al. (2024a); Zheng et al. (2024)

### 2.1 M6A

M6A refers to the methylation at the nitrogen atom in the sixth position of adenosine, which is the most abundant internal modification in eukaryotic messenger RNA (mRNA). It is enriched near the stop codon, within the 3′UTR, and across long exonic regions (Wang et al., 2022; Wang Y. et al., 2024). Moreover, m6A is widely present in non-coding RNA (ncRNA), regulating the stability and degradation of microRNA (miRNA), long non-coding RNA (lncRNA), and circular RNA (circRNA), which in turn affects various important biological processes, such as tumor progression, immune evasion and drug resistance (Li R. et al., 2024). This modification is dynamically reversible, with the addition, removal and recognition jointly regulated by “Writers,” “Erasers” and “Readers” (Wei et al., 2025).

The m6A methylation modification is carried out by “Writers,” which primarily involves the precise catalysis of RNA substrates by the methyltransferase complex (MTC). Its core catalytic unit is the heterodimer formed by methyltransferase-like 3 (METTL3) and methyltransferase-like 14 (METTL14) (Dou et al., 2023). Wilms’ tumor 1-associating protein (WTAP) is responsible for directing the complex to localize within nuclear speckles and facilitating its specific binding to target mRNA (Huang et al., 2022). Vir-like m6A methyltransferase-associated (VIRMA) contributes to the region-specific distribution of m6A within the 3′UTR and near the stop codon of mRNA by recruiting the components of the catalytic core (Yue et al., 2018). Furthermore, zinc finger CCCH domain protein 13 (ZC3H13) regulates the nuclear localization of the complex (Wen et al., 2018), while RNA binding motif protein 15 (RBM15) and its paralog RBM15 B enhance its functional specificity by mediating recognition of particular RNA sequences (Patil et al., 2016). METTL16 is an independent, important m6A methyltransferase that primarily catalyzes the m6A modification of U6 small nuclear RNA (snRNA) and acts on a range of precursor mRNAs (pre-mRNAs) and ncRNAs (Warda et al., 2017). Zinc finger CCHC domain protein 4 (ZCCHC4) is also independent, as it specifically catalyzes m6A modification at highly conserved sites on 28S ribosomal RNA (rRNA), thereby directly regulating protein synthesis (Pinto et al., 2020).

The dynamic reversibility of the m6A modification is determined by its key demethylases. Currently, the primary demethylases responsible for m6A are Fat Mass and Obesity Associated Protein (FTO) and AlkB Homolog 5 (ALKBH5). Among them, FTO was the first to be discovered and plays a crucial role in regulating energy metabolism, weight control, and tumor development (Shen et al., 2022; You et al., 2022). Meanwhile, ALKBH5 exhibits high specificity for m6A modification sites. It is predominantly localized in the nucleus and mediates m6A demethylation, thereby directly regulating mRNA splicing, nuclear export, stability, and translation post-transcription (Shen et al., 2022).

The diverse functional roles of m6A modification are primarily mediated by m6A-binding proteins. Members of the YTH family (such as YTHDF1/2/3 and YTHDC1/2) regulate mRNA translation, degradation, splicing, and nuclear export via direct recognition of m6A (Deng et al., 2023). Heterogeneous nuclear ribonucleoproteins (hnRNPs), such as hnRNP C, hnRNP G and HNRNPA2B1, are vital in post-transcriptional regulation, finely regulating pre-mRNA splicing by competing for RNA binding sites or recruiting helper factors. HNRNPA2B1 can also function as an m6A reader, directly recognizing and promoting the maturation of primary miRNA (pri-miRNA) (Li R. et al., 2024). Insulin-like growth factor 2 mRNA-binding proteins (IGF2BP1/2/3) boost the stability and translation of target mRNA by recognizing m6A (Deng et al., 2023). Additionally, eukaryotic translation initiation factor (eIF3) can directly bind m6A at the 5′UTR to initiate translation without relying on the cap (Lacerda et al., 2017). Fragile X Messenger Ribonucleoprotein 1 (FMRP) can also play a role in the precise regulation of neuronal translation by binding to m6A (Hsu et al., 2019).

### 2.2 M5C

M5C is a methylation modification at the fifth carbon atom of the cytosine base in RNA molecules. This modification is widely found in mRNA, transfer RNA (tRNA), rRNA, and other ncRNAs (Lu et al., 2024; Chen YS. et al., 2021; Wang J. et al., 2025). In mRNA, the m5C modification is most commonly found in the 3′UTR but can also be present within the 5′UTR and coding region (CDS), dynamically influences RNA expression, stability and translation efficiency (Chen YS. et al., 2021; Wang J. et al., 2025). In tRNA and rRNA, the m5C modification maintains their structural stability and functional integrity (Lu et al., 2024; Li et al., 2019). In lncRNA, the m5C modification also affects stability and various regulatory functions (Shi et al., 2020). Similar to m6A, the m5C modification is regulated by three classes of factors: “Writers,” “Erasers” and “Readers”.

The m5C modification on RNA is catalyzed by the NOP2/Sun RNA methyltransferase family (NSUN1-7) and specific methyltransferases including DNA methyltransferase homolog 2/tRNA aspartic acid methyltransferase 1 (DNMT2/TRDMT1) (Li M. et al., 2022). Different methyltransferases have distinct target specificities and functions: NSUN1 and NSUN5 primarily act on 28S rRNA, participating in the regulation of cell proliferation; NSUN2 has a broad range of targets and plays important roles in cell growth, metabolism and tumor cell migration; NSUN3 specifically modifies mitochondrial tRNA (mt-tRNA), is upregulated in various cancers and is also associated with immune cell infiltration; NSUN4 is mainly responsible for the methylation of small rRNA subunits, impacting mitochondrial protein synthesis (Yang et al., 2023; Li M. et al., 2022). DNMT2/TRDMT1 maintains tRNA stability and regulates the translation of specific mRNAs by methylating the anticodon loop of tRNA (Lu et al., 2024). Studies have also revealed that it participates in the regulation of mRNA methylation, thereby influencing cell migration and invasion (Li M. et al., 2022). Recent studies further reported that METTL17, as a key mitochondrial m5C methyltransferase, regulates the translation of mt-RNA and cellular energy metabolism through these modifications, thereby influencing processes such as tumorigenesis and a form of regulated cell death known as ferroptosis (Li H. et al., 2024).

Demethylating m5C is a process that involves two types of “Erasers”: the Ten-Eleven Translocation family enzymes (such as TET1 and TET2) and the AlkB homolog 1 (ALKBH1). They are both α-ketoglutarate/Fe^2+^-dependent dioxygenases, but they catalyze distinct reactions. TET family enzymes mainly convert m5C into 5-hydroxymethylcytosine (hm5C), leading to accumulation of this product (Fu et al., 2014; He et al., 2011). On the other hand, ALKBH1 oxidizes hm5C and turns it into 5-Formylcytidine (f5C) (Arguello et al., 2022). Both contribute to RNA m5C demethylation, influencing processes such as gene expression, RNA stability, and RNA translation.

The main “Readers” of m5C modifications include the ALY/REF export factor (ALYREF) and the Y-box binding protein 1 (YBX1). ALYREF (also known as THOC4) regulates mRNA nuclear export by recognizing particular sequence motifs (Fan et al., 2019). YBX1 binds to m5C via its cold shock domain (CSD) and is involved in essential cellular processes like RNA metabolism and DNA repair (Wang J. et al., 2025). FMRP is thought to be a non-classical m5C reader, contributing to the fine-tune of translation in neurons by recognizing specific m5C modifications on target mRNAs (Zhao T. et al., 2025).

### 2.3 M1A

M1A is a methylation at the N1 position of adenosine, which is positively charged under normal physiological conditions and significantly affects the local structure and function of RNA. It is found in mRNA, tRNA, rRNA, and mitochondrial transcripts (Zhang and Jia, 2018). In mRNA, m1A is primarily enriched in the 5′UTR region, where it disrupts Watson-Crick base pairing, interferes with reverse transcription processes, and participates in translation regulation. Abnormal modifications of m1A have been linked to the proliferation, invasion and immune evasion of various tumors (Wang MK. et al., 2023; Wang X. et al., 2024). In tRNA and rRNA, m1A utilizes its positive charge to regulate the secondary structure of RNA and RNA-protein interactions, which are key for maintaining the proper conformation of tRNA and for protein synthesis (Pajdzik et al., 2024; Xiong et al., 2018). The m1A in the CDS region of mt-mRNA can slow down translation elongation (Zhang and Jia, 2018; Safra et al., 2017). The m1A modification is dynamically reversible, and its “Writers” and “Erasers” have been identified; however, the existence of specific reader proteins is still a mystery, which leaves the entire regulatory process incompletely understood.

The widely recognized core m1A “Writers” mainly include the tRNA methyltransferase six and 61A (TRMT6–TRMT61A) complex and the TRMT61 B-TRMT10C complex. TRMT6-TRMT61A is found in the nucleus and catalyzes the m1A modification at position 58 of tRNA, a modification that has been highly conserved throughout evolution and plays a key role in maintaining the structural stability and biological functions of tRNA (Pajdzik et al., 2024; Liu et al., 2022a). On the other hand, the TRMT61 B-TRMT10C complex in the mitochondria maintains the stability and function of mt-tRNA by catalyzing the m1A modification, which is essential for effectively translating its genome (Smoczynski et al., 2024). Additionally, the nucleolar ribosomal RNA processing protein 8 (RRP8) catalyzes m1A modification of 28S rRNA, ensuring that functional ribosomes mature correctly (Zhu et al., 2018).

M1A demethylases are mainly composed of the ALKBH family, with ALKBH3 and ALKBH1 as the core members. ALKBH3 catalyzes the demethylation of m1A modifications in tRNA and mRNA, playing a major role in regulating RNA metabolism and function (Yang et al., 2023; Li et al., 2016). ALKBH1 primarily influences translation and protein synthesis efficiency by regulating functional tRNA production (Liu et al., 2016).

Currently, the specific “Readers” for m1A modifications are not well-defined, and this remains a subject of considerable debate (Li et al., 2016). Although some studies suggest that YTHDF1/2/3 and YTHDC1 may possess this function (Dai et al., 2023; Dai et al., 2018), this conclusion has not gained wide consensus. There is still no solid evidence that these proteins specifically recognize m1A. Therefore, there is a pressing need to confirm their functions in living organisms.

### 2.4 M7G

M7G is a methylation modification occurring at the nitrogen atom in the seventh position of guanine within RNA, widely distributed at two functionally crucial sites in RNA molecules. Its most well-known site is at the 5′cap structure of mRNA (m7GpppN), which plays a crucial role in maintaining mRNA stability, preventing degradation, promoting mRNA export, and initiating cap-dependent translation (Furuichi et al., 1977; Mamot et al., 2017; Zhang M. et al., 2023). On the other hand, m7G is also found as an internal modification in mRNA, tRNA, rRNA, and pri-miRNA. Its functions include regulating mRNA translation and stability, maintaining tRNA structural stability, promoting 18S rRNA maturation, and mediating miRNA biogenesis. These roles highlight its diverse regulatory functions in RNA metabolism (Luo et al., 2022; Zhao Z. et al., 2023). Currently, while m7G “Writers” and “Readers” have been well characterized, specific m7G “Erasers” remain unidentified, leaving the regulatory cycle incomplete.

The formation of the m7G modification is catalyzed by specific methyltransferase complexes. The m7G cap at the 5′end of mRNA is formed in the nucleus by the RNA guanine-7 methyltransferase (RNMT)–RNMT-activating miniprotein (RAM) complex (Bueren-Calabuig et al., 2019). The m7G modifications found in tRNA, miRNA and mRNA are mainly catalyzed by the METTL1–WD repeat-containing protein 4 (WDR4) complex, which is overexpressed in various gastrointestinal tumors and significantly boosts tRNA stability while increasing the translation efficiency of cancer-promoting transcripts, driving tumor growth and invasion (Ruiz-Arroyo et al., 2023). Additionally, the m7G modification in rRNA is primarily catalyzed by the Williams-Beuren syndrome critical region protein 22 (WBSCR22)–TRMT112 complex, which is crucial for the normal maturation and function of ribosomes (Zhang M. et al., 2023).

To date, no clear and established m7G “Erasers” have been found, and their dynamic regulation might be partly achieved through RNA degradation facilitated by decapping enzymes such as Nudix Hydrolase 16 (NUDT16) (Taylor and Peculis, 2008). As for function, the m7G modification is mediated by its specific m7G “Readers”. The m7G cap at the 5′end of mRNA is mainly recognized and bound by the eukaryotic translation initiation factor 4E (eIF4E) and the cap-binding complex (CBC), which initiate translation and mediate mRNA processing and export from the nucleus (Sato and Maquat, 2009; Mars et al., 2021). Furthermore, Quaking proteins (such as QKI5, QKI6, and QKI7) can recognize m7G modifications and regulate mRNA stability and translation efficiency under stress conditions (Han M. et al., 2024; Zheng et al.).

## 3 Role of RNA methylation in reprogramming the immune microenvironment of gastrointestinal tumors

Increasing evidence suggests that RNA methylation regulates the immune microenvironment of gastrointestinal tumors through three interconnected axes. First, it can directly reshape the fate and function of immune cells in the TIME, including T cell differentiation into effector cells and exhaustion phenotypes, the growth of MDSCs, and the M1/M2 polarization balance of TAMs (Quan et al., 2021; Mehdi and Rabbani, 2021). Simultaneously, modified RNA from tumor cells can also influence immune cells infiltration via intercellular communication (Hu et al., 2024). Second, RNA methylation regulates immunogenicity within tumor cells: on the one hand, by changing the production of tumor-associated antigens/neopeptides and the efficiency of major histocompatibility complex (MHC) presentation, influencing immunogenic cell death and amplifying interferon signaling to shape its immunogenicity; on the other hand, by directly regulating the expression of immune checkpoint molecules such as Programmed Death-Ligand 1 (PD-L1) at the post-transcriptional level, driving immune evasion and ultimately determining whether the tumor can be effectively cleared by the immune system (Li et al., 2024c). Third, RNA methylation drives immunometabolic reprogramming. In the context of this review, this term refers to the RNA methylation-mediated systematic rewiring of metabolic pathways—such as glycolysis, lipid metabolism, and amino acid utilization—within both tumor cells and immune cells, which collectively shape the functional state of the TIME. This process often creates an inhibitory metabolic environment marked by the accumulation of lactate and lipids; furthermore, metabolic intermediates can feed back to modulate the levels of methyl donors and the activity of demethylases, forming a “metabolism-modification-immunity” feedback loop (Li et al., 2024c; Zhao L. et al., 2025). Given the different immune backgrounds and metabolic characteristics of various gastrointestinal tumors, the specific regulatory patterns and key targets of these three axes differ across cancer types. The following sections will discuss the detailed cancer type-specific mechanisms and clinical significance of RNA methylation in HCC, CRC, GC, PDAC, and ESCA (Table 2).

**TABLE 2:** Roles of RNA methylation in immune responses of gastrointestinal tumors.

Cancer type	Methylation	Regulator	Regulatory pathway	Biological function	Impact	References
HCC	m6A	METTL3/14	L1CAM-AS1-RAN axis	Induces TAMs M2 polarization	Promotes immunosuppression and HCC progression	Wang et al. (2025b)
		FTO	FTO/m6A/GPNMB axis	Inhibits CD8^+^ T-cell activation	Promotes immune evasion and tumor progression	Chen et al. (2024)
		YTHDF1	EZH2-IL-6 signaling	Induces CD8^+^ T cell exhaustion	Promotes tumor progression and immunotherapy resistance	Wang et al. (2023b)
		YTHDF2	ETV5/PD-L1/VEGFA axis	Upregulates PD-L1 and VEGFA.	Drives immune evasion and angiogenesis	Wen et al. (2024)
		IGF2BP2/3	KLF16-C12orf49-PD-L1 axis	Upregulates PD-L1	Promotes immune evasion	Chen et al. (2025)
	m5C	NSUN2	NSUN2-SOAT2	Inhibits CD8^+^ T-cell activation	Drives immune evasion and tumor growth	Jiang et al. (2025a)
	m7G	METTL1	METTL1-TGF-β2-PMN-MDSC axis	Drives PMN-MDSCs accumulation	Promotes tumor progression	Zeng et al. (2023)
CRC	m6A	METTL14	H3K18la-METTL14-m6A axis	Induces TAMs M2 polarization	Promotes tumour progression and immunosuppression	Liu et al. (2025a)
		METTL3	m6A-BHLHE41-CXCL1/CXCR2 axis	Recruits MDSCs to inhibit CD8 T cells	Promotes CRC progression	Chen et al. (2022)
		METTL3	miR-326/miR-330-5p/PGAM1 axis	Expands Treg population	Leads to anti-CTLA-4 resistance	Liu et al. (2022b)
		ALKBH5	ALKBH5-m6A-AXIN2-Wnt-DKK1 axis	Recruits MDSCs to inhibit CD8 T cells	Drives immunosuppression	Zhai et al. (2023)
		YTHDF1	m6A-p65-CXCL1/CXCR2 axis	Recruits MDSCs to inhibit CD8 T cells	Promotes CRC progression	Bao et al. (2023)
		IGF2BP1	IGF2BP1-PD-L1	Inhibit CD8 T cells	Accelerates immune evasion	Peng et al. (2024)
	m7G	METTL1	METTL1/PKM2/H3K9la axis	Activates CD155 expression	Induces immune evasion	Wang et al. (2024c)
GC	m6A	METTL3	3’tRF-AlaAGC/PTBP1/PKM2/LA/Treg-MCT1/Treg-PD1 axis	Expands Treg population	Leads to TME disturbance and resistance to ICI therapy	Xu et al. (2025)
		FTO	FTO/NNMT axis	Promotes TAMs M2 polarization	Drives GC progression	Mak et al. (2024)
		ALKBH5	HSPA4/ALKBH5/CD58 axis	Activates the PD-1/PD-L1 pathway	Induces immune evasion	Suo et al. (2024)
		IGF2BP2	IGF2BP2/PD-L1 axis	Promotes T cell exhaustion	Induces immune evasion	Li et al. (2024d)
	m1A	-	NFAT/TOX signaling	Promotes T cell exhaustion	Leads to immunotherapy resistance	Yu et al. (2024)
PDAC	m6A	METTL3/IGF2BP2	circMYO1C/IGF2BP2/PD-L1	Upregulates PD-L1	Promotes immune evasion and tumor progression	Guan et al. (2023)
		METTL16	lactate-METTL16-CTCF axis	Impairs CD8^+^ T cell function	Leads to resistance to PD-1 therapy	Liu et al. (2025c)
		IGF2BP2	IGF2BP2/CSF1/CSF1R axis	Induces TAMs M2 polarization	Promotes tumor progression	Liu et al. (2026)
		IGF2BP2	IGF2BP2/SGMS2 axis	Upregulates PD-L1	Enhances immune evasion	Tang et al. (2025)
	m5C	ALYREF	ALYREF-JunD-SLC7A5 axis	Impairs CD8^+^ T cell function	Promotes immune evasion	Meng et al. (2024)
ESCA	m6A	METTL3/14	CXCL8-CXCR2 axis	Drives TAMs M2 polarization	Promotes tumor progression	Yan et al., (2025)
		METTL3/FTO/ALKBH5/IGF2BP2	SHMT2-m6A-MYC	Upregulates PD-L1	Regulates tumor progression and immune evasion	Qiao et al., (2023)

### 3.1 RNA methylation and HCC

#### 3.1.1 Role of m6A in HCC immune regulation

The liver itself is an immune-tolerant organ. In the context of chronic inflammation caused by persistent viral infection and liver cirrhosis, the TIME in HCC often exhibits characteristics such as T cell exhaustion, the accumulation of MDSCs, and impaired antigen presentation, which leads to the tumor becoming resistant to immunotherapy (Zheng and Tian, 2019). In recent years, RNA modifications, particularly m6A, have increasingly been recognized as key “switches” in regulating tumor immune responses. M6A significantly influences the immune landscape of HCC by altering the fate of immune cells, regulating tumor immune evasion, and reprogramming the metabolic state of the TME.

##### 3.1.1.1 M6A modulates immune cells

During the progression of HCC, the functional state of immune cells is crucial: effector T cells are responsible for directly attacking tumors, dendritic cells (DCs) play a central role in antigen presentation and immune activation, while myeloid-derived immune suppressor cells (MDICs) contribute to the formation of an immunosuppressive microenvironment. Recent evidence shows that the m6A modification carefully balances this immune response through three key processes.

T cell exhaustion is a key factor impacting the prognosis of HCC, closely related to tumor mutation burden (TMB) and tumor-infiltrating lymphocytes (TILs), and serves as one of the core mechanisms of tumor immune evasion (Chi et al., 2023; Zheng et al., 2017). Recently, researchers found that the m6A demethylase FTO helps stabilize the mRNA of the glycoprotein non-metastatic melanoma protein B (GPNMB), facilitating the packaging of its encoded protein into small extracellular vesicles (sEVs), which then attaches to the syndecan-4 (SDC4) receptor on the surface of CD8^+^ T cells, inhibiting T cell activation (Chen et al., 2024). Therefore, targeting the m6A regulatory network holds promise as a new strategy to reverse T cell exhaustion and enhance the efficacy of HCC immunotherapy. Notably, in the HCC microenvironment, the regulatory scope of m6A modifications now includes core immune sentinels such as DCs. Studies have shown that the m6A reader YTHDF1 enhances the capacity of DCs to degrade antigens by recognizing and promoting the translation of lysosomal protease mRNA, thereby inhibiting the cross-presentation of neoantigen peptide-MHC class I complexes (pMHC I). Meanwhile, the absence of YTHDF1 may reduce antigen degradation and enhance cross-presentation efficiency, which then activates long-lasting neoantigen-specific CD8^+^ T cell anti-tumor immunity and synergizes with PD-L1 blockade therapy (Han et al., 2019). However, the potential trade-off between enhanced cross-presentation and autoimmune risk, following YTHDF1 inhibition, warrants careful consideration in the development of therapeutics.

In addition, m6A modifications play a critical role in establishing the suppressive microenvironment of HCC by reprogramming MDICs. In terms of TAMs, studies show that histone H3 trimethylation at lysine-36 (H3K36me3) recruits the METTL3/14 complex to catalyze m6A modification on the L1 cell adhesion molecule antisense RNA 1 (L1CAM-AS1) transcript. L1CAM-AS1 inhibits RAN (a member of the RAS oncogene family) ubiquitin-mediated degradation, thereby stabilizing the RAN protein. This activates the nuclear factor-kappa B (NF-κB) signaling pathway, which in turn drives the M2 polarization of TAMs via a chemokine (C-C motif) ligand 2 (CCL2)/ligand 5 (CCL5) positive feedback loop (Wang T. et al., 2025). Targeting L1CAM-AS1 has been shown to enhance the efficacy of PD-1 inhibitors, hinting at its potential as a target for combination immunotherapy. In the case of MDSCs, the m6A reader YTHDF1 promotes the occurrence and development of non-alcoholic steatohepatitis (NASH)-related HCC through the Enhancer of Zeste Homolog 2 (EZH2)-Interleukin-6 (IL-6) signaling axis, which can recruit and activate MDSCs, leading to CD8^+^ T cell functional exhaustion (Wang L. et al., 2023). This reveals a novel mechanism by which m6A regulates immune evasion in NASH-related HCC.

##### 3.1.1.2 M6A regulates immune evasion

Recent research has shown that the m6A modification, as a key epitranscriptomic regulator, plays a central role in the context of HCC immune evasion by precisely regulating the expression of immune-related molecules such as PD-L1. Its regulatory network primarily includes the tumor cells and pathogenic microorganisms.

At the tumor cell level, YTHDF2 promotes the translation of ETS variant transcription factor 5 (ETV5) mRNA by recognizing it, thereby upregulating PD-L1 and vascular endothelial growth factor A (VEGFA), leading to immune evasion and angiogenesis in HCC (Wen et al., 2024). Targeting YTHDF2 can effectively inhibit this process, which suggests that it could be a potential therapeutic target. At the pathogen and microbiome level, the m6A modification network closely interacts with various external factors, jointly shaping the immunosuppressive microenvironment of HCC. In hepatitis B virus (HBV)-related HCC, Hepatitis B virus X protein (HBx) enhances the stability of Kruppel-like factor 16 (KLF16) mRNA through the m6A-IGF2BP2/3 axis, which then transcriptionally activates chromosome 12 open reading frame 49 (C12orf49). This encodes a protein that competes with speckle-type POZ protein (SPOP), blocking the pathway that degrades PD-L1 through ubiquitination, ultimately leading to immune evasion due to PD-L1 accumulation (Chen et al., 2025). In addition to viral factors, lipopolysaccharides (LPS) derived from the gut microbiome can increase METTL14 expression, which promotes the m6A methylation of lncRNA MIR155 host gene (MIR155HG), enhancing its stability through ELAV like RNA binding protein 1 (ELAVL1). The highly expressed MIR155HG acts as a competing endogenous RNA (ceRNA) that binds to miR-223, lifting its inhibition on signal transducer and activator of transcription 1 (STAT1), ultimately upregulating PD-L1 expression and creating a regulatory pathway that connects the “microbe-m6A-immune checkpoint” (Peng et al., 2022). These findings clearly show the central role of m6A modification in integrating pathogenic signals and microbiome factors and reprogramming the TIME, offering new insights into how different layers regulate immune evasion in HCC.

##### 3.1.1.3 M6A reprograms tumor immune metabolism

In the HCC microenvironment, one of the key factors driving tumor immune evasion is immune metabolic reprogramming. Recent research has shown that the m6A modification is key in the metabolic reprogramming of HCC by carefully controlling the related metabolic enzymes.

In the process of lactate metabolism in HCC, tumor cells transfer SLC16A1-AS1 (an antisense lncRNA) to macrophages via exosomes. This molecule enhances the stability of SLC16A1 mRNA by binding to HNRNPA1, enhancing lactate influx into the cells and activating the downstream c-Raf/extracellular signal-regulated kinase (ERK) signaling pathway, which causes macrophages to shift towards the M2 phenotype. Polarized M2 macrophages further secrete IL-6, which activates the STAT3-METTL3 signaling axis in HCC cells, enhancing the stability of SLC16A1-AS1 itself through the m6A modification, thus forming a self-reinforcing feedback loop that drives tumor malignant progression (Hu et al., 2024). This mechanism suggests that targeting SLC16A1-AS1 could effectively disrupt these harmful metabolic-immune cycles. In the cholesterol metabolism process of HCC, METTL3 enhances the translation efficiency of sterol regulatory element-binding protein (SREBP) cleavage-activating protein (SCAP) mRNA through the m6A modification, thereby activating the cholesterol biosynthesis pathway and leading to an unusual buildup of cholesterol and cholesterol esters in the TME. This metabolic process directly reduces granzyme B (GZMB+) and interferon gamma-positive (IFN-γ+) CD8^+^ T cell infiltration, thereby weakening their anti-tumor immune function (Pan et al., 2023). This study shows how cholesterol buildup helps HCC evade the immune system from the perspective of metabolic-immune interaction. Furthermore, exosomal circPETH derived from TAMs encodes a peptide circPETH-147aa in an m6A-dependent manner, driving comprehensive metabolic reprogramming. This peptide boosts the pyruvate kinase M2 (PKM2)-dependent phosphorylation of ALDOA-S36 via the MEG pocket, enhancing glycolysis and promoting tumor progression, while also leading to disruptions in amino acid metabolism due to the role of human antigen R (HuR) in stabilizing solute carrier family 43 member 2 (SLC43A) mRNA, resulting in the functional exhaustion of CD8^+^ T cells. This complex metabolic-immune suppression can be effectively reversed by small molecule dephosphorylated trihydroxy alcohol via targeting the MEG domain of this peptide and boosting the effectiveness of PD-1 inhibitors (Lan et al., 2025).

#### 3.1.2 Role of other RNA methylations in immune regulation of HCC

Compared to the well-studied m6A modification, there is still relatively little evidence linking other types of RNA methylation modifications to immune regulation in HCC, and these research efforts are quite fragmented. For m5C, the methyltransferase NSUN2 has been shown to enhance the stability of sterol O-acyltransferase 2 (SOAT2) mRNA by mediating its m5C modification, which helps reprogram cholesterol ester metabolism in tumor cells, ultimately leading to immune evasion by inhibiting the activation and cytotoxic function of CD8^+^ T cells (Jiang J. et al., 2025). Studies on m7G modifications found that radiofrequency ablation therapy can boost the expression of the methyltransferase METTL1, which then promotes the tRNA m7G modification and boosts the translation of transforming growth factor-beta 2 (TGF-β2), ultimately leading to the accumulation of polymorphonuclear myeloid-derived suppressor cells (PMN-MDSCs) in the TME and inhibits CD8^+^ T cell function. This research reveals the critical role of METTL1 in establishing an immunosuppressive microenvironment and suggests that targeting the METTL1–TGF-β2–PMN-MDSC axis could be a promising strategy for restoring anti-tumor immunity and preventing HCC recurrence after radiofrequency ablation (Zeng et al., 2023). Overall, further research is needed to reveal the specific roles and cross-talk mechanisms of these modifications in HCC immune evasion.

#### 3.1.3 RNA methylation-based immune prognostic models for HCC

In recent years, RNA methylation modifications represented by m6A have been shown to play a key role in shaping the immunosuppressive microenvironment of HCC through complex post-transcriptional regulatory networks. To translate this mechanism into clinically applicable tools, researchers are working to construct immune prognostic models based on RNA methylation features. At the level of m6A modification, multiple studies have revealed the heterogeneity of its regulatory network, and the molecular subtyping and scoring systems built on this can effectively assess the “cold” and “hot” characteristics of the TIME as well as immunotherapy sensitivity (Wang H. et al., 2025; Xie et al., 2022; Xu et al., 2024). In the field of m7G modification, research has developed a more targeted mRNA/lncRNA risk model, offering specialized tools for assessing HCC progression and immune evasion (Li R. et al., 2023). Furthermore, integrative analyses of multiple modifications have revealed more complex regulatory networks. The epigenetic and epitranscriptomic module eigengene (EME) scoring system developed from the synergistic effects of m5C and m6A is closely related to potential external and internal immune evasion mechanisms (Tian et al., 2023). It is worth noting that research in this area is still in the early stages, and the practical value of existing models in clinical translation needs to be further validated through in-depth functional experiments and large-scale prospective cohort studies.

#### 3.1.4 Role of RNA methylation in HCC: summary

**FIGURE 1:**
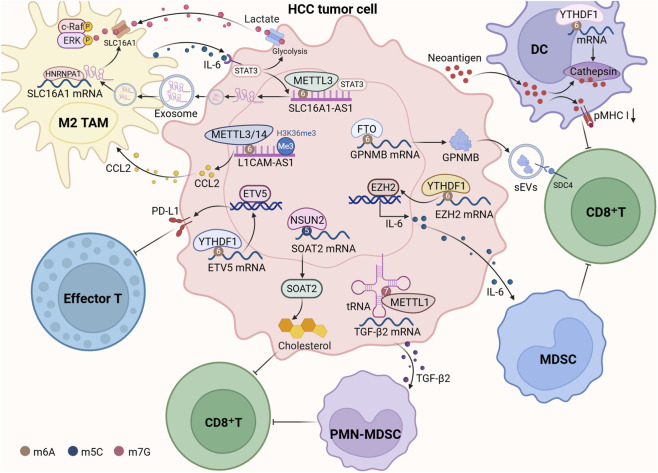
Regulatory mechanism of RNA methylation in the immune microenvironment of HCC. At the level of immune cells, FTO delivers GPNMB through sEVs (small extracellular vesicles) to inhibit CD8^+^ T cell function; YTHDF1 inhibits cross-presentation by enhancing antigen degradation in DCs; meanwhile, m6A drives TAM polarization towards M2 by stabilizing molecules such as L1CAM-AS1 and recruits activated MDSCs via the YTHDF1-EZH2-IL-6 axis. At the level of immune escape, YTHDF2 acts as upstream regulators that drive PD-L1 expression. At the level of immune metabolism, m6A mediates the reprogramming of lactate and cholesterol metabolism, synergistically promoting TAM polarization and T cell function inhibition. In addition, modifications such as m5C and m7G also participate in immune suppression via affecting metabolism or activating MDSCs.

In summary, the RNA methylation landscape in HCC is characterized by a distinctive “pathogen-metabolism-immune” integrative network that reflects liver-specific etiology and physiological function (Figure 1). Specifically, m6A modification forms a hallmark “pathogen-epigenetic-checkpoint” axis that converts external cues—such as HBV infection and gut-derived LPS—into an intrinsic immune evasion program driven by PD-L1 stabilization. Moreover, leveraging the liver’s role as a metabolic hub, RNA methylation (m6A/m5C) orchestrates a “cholesterol-immune” metabolic axis, in which dysregulated lipid metabolism contributes to T cell exhaustion—a feature particularly pronounced in metabolic dysfunction-associated HCC. By mechanistically bridging chronic inflammation, metabolic reprogramming, and the TME, these liver-specific regulatory axes define the immunosuppressive landscape unique to HCC, thereby distinguishing it from other gastrointestinal tumors.

### 3.2 RNA methylation and CRC

#### 3.2.1 Role of m6A in immune regulation of CRC

CRC is a type of cancer that is prevalent worldwide, with different molecular subtypes showing significant differences in clinical presentation and treatment response. In recent years, immune checkpoint inhibitors (ICIs) have been shown to be effective in certain CRC patients. However, they are ineffective against subtypes characterized by proficient mismatch repair (pMMR), microsatellite stable (MSS), or microsatellite instability-low (MSI-L). This highlights the challenging clinical scenario for pMMR-MSS/MSI-L CRC due to its “immune cold” microenvironment (Ganesh et al., 2019). In this context, the m6A modification is a crucial mechanism regulating RNA and has been shown to play a significant role in immune responses and tumor immune evasion, which means that it may be key in shaping the immune environment of pMMR-MSS/MSI-L CRC. Therefore, looking closely at how the m6A modification regulates immunity in CRC provides important insights, which could help us understand how CRC resists immune attacks and lead to better combination therapies.

##### 3.2.1.1 M6A modulates immune cells

In CRC, the m6A modification is a key mechanism of epitranscriptional regulation: it drives the formation of an “immune cold” microenvironment by directly inhibiting the function of CD8^+^ T cells, systemically remodeling MDSCs, and reshaping the chemokine network.

CD8^+^ T cells, as core executors of anti-tumor immunity, exhibit poor infiltration and functional exhaustion in the CRC microenvironment, which are key contributors to immunotherapy resistance. Specifically, the m6A modification that METTL14 mediates in TAMs regulates the expression of Epstein-Barr virus-induced protein 3 (EBI3), leading to CD8^+^ T cell dysfunction, while EBI3 blockade may reverse this inhibitory effect (Dong et al., 2021). When it comes to MDSC recruitment, ALKBH5 promotes the demethylation of axis inhibition protein 2 (AXIN2) mRNA, blocking its binding to IGF2BP1, thereby promoting AXIN2 degradation and activating the Wnt/β-catenin signaling pathway, which then increases Dickkopf-related protein 1 (DKK1) expression. This process ultimately facilitates MDSC recruitment and inhibits CD8^+^ T cell function, driving immunotherapy resistance (Zhai et al., 2023). This finding provides a basis for new combination immunotherapy strategies targeting the m6A-myeloid immune axis. Chemokines also regulate how immune cells function in the TME. METTL3 boosts BHLHE41 expression in an m6A-dependent manner, enhancing the transcription of C-X-C motif chemokine ligand 1 (CXCL1) (Chen et al., 2022); simultaneously, YTHDF1 recognizes p65 mRNA and promotes its translation, activating the CXCL1-CXCR2 chemokine axis (Bao et al., 2023). These pathways together guide MDSCs to the TME, thereby inhibiting T cell function and reducing the effectiveness of immunotherapy. In the future, treatments might combine blocking the CXCL1-CXCR2 axis with ICIs to reverse the immunosuppressive microenvironment and overcome resistance to immunotherapy.

##### 3.2.1.2 M6A regulates immune evasion

Research has shown that the m6A modification plays a role in immune evasion in CRC by regulating PD-L1 expression. IGF2BP1 has been shown to recognize and bind to the 3′-UTR of PD-L1 mRNA in an m6A-dependent manner, enhancing its stability and promoting PD-L1 protein expression, which in turn reduces the tumor-killing ability of CD8^+^ T cells (Peng et al., 2024). Additionally, *Fusobacterium nucleatum* has also been shown to enhance the stability of IFIT1 mRNA by activating METTL3/METTL14 to catalyze the m6A modification, which is recognized by IGF2BP2/3. The upregulated IFIT1 protein inhibits the ubiquitination and degradation of PD-L1, thereby increasing PD-L1 level and mediating immune evasion in CRC (Gao et al., 2023). Therefore, targeting the m6A regulatory axis in combination with ICIs could work together to reverse PD-L1 upregulation and boost the effectiveness of immunotherapy.

##### 3.2.1.3 M6A reprograms tumor immune metabolism

The onset and development of CRC are closely related to changes in immune metabolism in the TME. In terms of glycolysis, the m6A modification mediated by METTL3 stabilizes circQSOX1 (competing endogenous RNA) together with IGF2BP2. This complex boosts phosphoglycerate mutase 1 (PGAM1) expression by soaking up miR-326/miR-330-5p, thereby promoting the glycolytic pathway and enhancing the immunosuppressive function of Treg cells, which ultimately induces resistance to anti-cytotoxic T lymphocyte antigen-4 (CTLA-4) therapy (Liu Z. et al., 2022). Therefore, targeting this pathway can weaken Treg function, thereby enhancing anti-tumor immunity. When it comes to amino acid metabolism, methionine metabolism facilitates the m6A modification of PD-L1 and V-domain Ig suppressor of T cell activation (VISTA) mRNA through the generation of S-adenosylmethionine (SAM), with YTHDF1 boosting its translation by recognizing this modification. This results in an elevated level of immune checkpoint molecules and subsequently inhibits the activity of CD8^+^ T cells (Li T. et al., 2023). In terms of fatty acid metabolism, the buildup of lactate in the TME can increase METTL14 by causing H3K18 lactylation, which boosts the immune regulatory molecule V‐set immunoglobulin‐domain‐containing 4 (VSIG4) in an m6A-dependent way, encouraging macrophages to undergo fatty acid oxidation (FAO) by activating the JAK2/STAT3 signaling pathway, promoting their polarization to the M2 type, thereby creating an immunosuppressive environment and reducing the effectiveness of anti-PD-1 therapy (Liu J. et al., 2025). In summary, this metabolic system indicates that targeting m6A-mediated immune metabolism changes could offer novel strategies to tackle CRC immunotherapy resistance.

#### 3.2.2 Role of other RNA methylations in immune regulation of CRC

Apart from m6A, research on the role of other RNA methylation modifications in CRC immune regulation is starting to gain some ground, while it is still relatively limited. Existing evidence suggests that the methyltransferase METTL1 enhances the stability of PKM mRNA by adding a m7G modification, promoting the expression of the PKM2 protein, which creates a positive feedback loop involving glycolysis, H3K9 lactylation, and METTL1 transcription. Meanwhile, the PKM2 translocated into the nucleus further activates the immune checkpoint CD155, working together to drive metabolic reprogramming and immune evasion in CRC (Wang F. et al., 2024). On the other hand, the tRNA-derived fragment tRF-3022b is known to target the galectin-1 (LGALS1) and macrophage migration inhibitory factor (MIF) signaling axis, regulating M2 macrophage polarization. Research has also found that the production of tRFs might be dynamically controlled by the demethylase ALKBH3 and the methyltransferase DNMT2 (Lu et al., 2022). This suggests that m1A and m5C modifications might affect the stability of tRNA and how it is shaped, influencing the efficiency and type of tRF produced when it is cut by nucleases. From this, it can be inferred that RNA methylations (such as m1A and m5C) may serve as upstream regulators in the tRF biogenesis, playing a role in the remodeling of the immune microenvironment and the metabolic regulation in CRC.

However, evidence regarding other RNA modifications in CRC immunoregulation is fragmented and occasionally contradictory. The m7G modification mediated by METTL1 appears to promote immune suppression, but whether this effect is consistent across various CRC models or modulated by tumor stage and genetic background has yet to be elucidated.

#### 3.2.3 RNA methylation-based immune prognostic models for CRC

Currently, there are scoring models based on RNA methylation modifications such as m6A, m5C and m1A, aiming to provide new molecular tools for stratifying and accurately predicting prognosis in CRC immunotherapy. Relevant studies consistently indicate that the low methylation scores are closely associated with an “immune hot” phenotype, which is characterized by higher TMB, increased expression of immune checkpoint molecules, and better responses to immunotherapy (Chong et al., 2021; Zhang R. et al., 2023; Gao et al., 2021). Furthermore, integrated composite scoring systems that incorporate multiple modification types (such as the WM_Score) have shown superior stability and predictive efficacy, promising more accurate immune microenvironment classification and assessment of treatment responses in clinical settings (Chen H. et al., 2021). Future research should explore the mechanisms linking dynamic changes in RNA methylation to responses to immunotherapy, in order to accelerate its practical application in targeted immunotherapeutic options for CRC.

#### 3.2.4 Roles of RNA modification in CRC: summary

**FIGURE 2:**
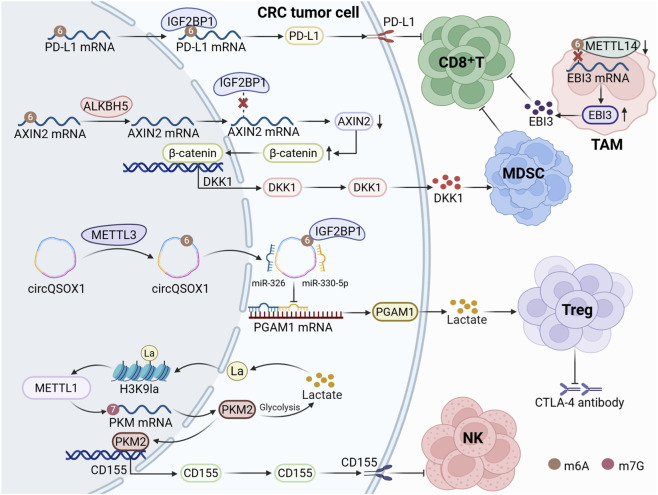
Regulatory mechanism of RNA methylation in the CRC immune microenvironment. At the level of immune cells, METTL14 inhibits CD8^+^ T cell function through the EBI3 axis, while ALKBH5 promotes MDSC recruitment. At the level of immune evasion, IGF2BP1 acts as an upstream hub to regulate PD-L1 expression. At the metabolic level, m6A mediates the reprogramming of glycolysis, thereby enhancing the immunosuppressive function of Treg. In addition, the m7G methyltransferase METTL1 stabilizes PKM2 to form a metabolic-immune evasion positive feedback loop.

In summary, the RNA methylation network in CRC reshapes the “cold tumor” microenvironment through three specific axes (Figure 2). *Fusobacterium nucleatum* exploits m6A modification to stabilize PD-L1, thereby establishing a microbiota-driven mechanism of immune evasion. The CXCL1–MDSC chemotactic cascade constitutes an immune exclusion axis characteristic of MSS/pMMR CRC. Lactylation couples with RNA methylation to upregulate VSIG4 and CD155, forming a metabolic feedback loop that reinforces immune checkpoint expression. Together, these axes define a CRC-specific immunosuppressive landscape and provide precision targets for overcoming immunotherapy resistance in MSS CRC.

### 3.3 RNA methylation and GC

#### 3.3.1 Role of m6A in immune regulation of GC

GC is globally one of the most burdensome cancers. Despite continuous advancements in treatment options in recent years, the outlook for patients with advanced disease remains poor, mainly because of tumor heterogeneity, drug resistance and the tendency to metastasize, which are all closely linked to the highly suppressive TIME (Liu et al., 2022c; He et al., 2025). The main features of this microenvironment include T cell exhaustion and inadequate infiltration, especially seen as typical “immune desertification” in genomically stable (GS) and chromosomal instability (CIN) types, resulting in overall poor responses to immunotherapy (Yasuda and Wang, 2024). In this context, the m6A modification serves as an upstream regulatory hub influencing both tumor cells and immune cells, driving immune metabolic reprogramming, which together worsen this immunosuppressive state and play a crucial role in GC progression and treatment response.

##### 3.3.1.1 M6A modulates immune cells

In GC, the m6A modification plays a key role in T cell activation and exhaustion, as well as macrophage polarization. It is also involved in exosome-mediated communication between cells, which profoundly influences anti-tumor immune responses through a complex network.

In terms of regulating T cell function, Histone acetylation increases the expression of heat shock protein family A (Hsp70) member 4 (HSPA4), which lowers CD58 levels by enhancing the stability of the ALKBH5 protein in an m6A-dependent manner. Lower CD58 levels impair the cytotoxic function of CD8^+^ T cells and activate the PD-1/PD-L1 pathway, which ultimately facilitates immune evasion by cancer cells in GC (Suo et al., 2024). Therefore, targeting the m6A regulatory axis or combining it with immune checkpoint blockade in GC could open up new avenues to restore T cell function and overcome resistance to immunotherapy. In terms of regulating macrophage polarization, high levels of FTO in CAFs boost the stability and levels of nicotinamide N-methyltransferase (NNMT) mRNA by lowering its m6A methylation, which causes M2 macrophage polarization and promotes GC progression (Mak et al., 2024). In terms of mediating intercellular communication, the overexpression of METTL3 in GC cells boosts the production of the Ras-related protein Rab-27A (RAB27A) through YTHDF1-dependent m6A modification, helping to deliver the miRNA-17-92 cluster to peritoneal macrophages via exosomes. This cluster blocks SRC kinase signaling inhibitor 1 (SRCIN1), activating the SRC proto-oncogene-encoded, non-receptor tyrosine kinase (SRC) signaling pathway, upregulating IL-10 and tumor necrosis factor (TNF), and changing the immunosuppressive environment, which ultimately weakens T cell function and encourages peritoneal metastasis (Li et al., 2025). Importantly, while this study demonstrated the role of m6A-driven exosomal communication in peritoneal metastasis of GC, it remains unclear whether similar mechanisms operate in other metastatic sites such as the liver and lymph nodes.

##### 3.3.1.2 M6A regulates immune evasion

The m6A modification also influences the immune evasion of GC by regulating the expression of PD-L1. Recent studies have shown that circRHBDD1 stabilizes IGF2BP2 protein by competitively binding to IGF2BP2 and preventing its ubiquitin-mediated degradation by the E3 ligase TRIM25. Elevated levels of IGF2BP2 enhance PD-L1 mRNA stability in an m6A-dependent manner, upregulating its expression, which ultimately inhibits CD8^+^ T cell function and leads to immunotherapy resistance, thereby promoting immune evasion in GC (Li et al., 2024d). Developing intervention strategies that target key nodes in this network, such as IGF2BP2, could be a breakthrough in improving the efficacy of immunotherapy for GC.

##### 3.3.1.3 M6A reprograms tumor immune metabolism

Currently, few direct examples of m6A regulation in GC immune metabolism exist. This scarcity might be due to the complexity of immune metabolism research, which covers areas such as epitranscriptomics, cellular metabolism, and immunology, making it challenging to analyze the relevant mechanisms (Lu et al., 2025). Existing studies have revealed that METTL3 stabilizes the tRNA-derived fragment 3’tRF-AlaAGC in an m6A-dependent manner and further interacts with polypyrimidine tract-binding protein 1 (PTBP1) to promote glycolysis and lactate production in GC cells. The high lactate levels promote lactate uptake by Treg cells through monocarboxylate transporter 1 (MCT1), activating the nuclear translocation of nuclear factor of activated T-cells 1 (NFAT1), which increases PD-1 expression, thereby boosting Treg immunosuppressive function and disrupting the balance between Treg and CD8^+^ T cells. This process ultimately results in resistance to ICIs (Xu et al., 2025). Future research on how m6A regulates the GC immune metabolism network will provide a foundation for new metabolic-immunotherapy combination strategies.

#### 3.3.2 Role of other RNA methylation in immune regulation of GC

In recent years, breakthroughs have been made regarding modifications such as m1A and m7G in GC. Specifically, the m1A modification stabilizes the transcript of SFRP2, thereby activating the downstream NFAT1/2-TOX signaling pathway. Activation of this pathway is a key driver of T cell differentiation into a functionally exhausted state, which ultimately leads to immune suppression and resistance to immunotherapy in GC (Liu et al., 2025b). As for the m7G modification, the methyltransferase METTL1 mediates the methylation of tRNA at the m7G position, which upregulates CTLA-4 and PD-1 expression, thereby inhibiting T cell proliferation and their anti-tumor functions in the TME, ultimately promoting immune evasion (Yu et al., 2024). Targeting METTL1 might reverse this state of immune suppression, offering a new potential target to boost the effectiveness of ICIs in GC.

#### 3.3.3 RNA methylation-based immune prognostic models for GC

Prognostic models based on the RNA modification profiles are becoming important tools for guiding targeted treatment for GC. Early studies have confirmed that low m6A scores are associated with an immune-activated microenvironment and better 5-year survival rates (Zhang et al., 2020). Further findings showed that low m7G scores are linked to MSI-H, high TMB and better responses to immunotherapy (Li X. et al., 2022). On this basis, several predictive models using RNA modification-related lncRNAs have been built that can effectively distinguish patient survival risks. These models also show potential for predicting responses to immunotherapy (Han et al., 2021; Zhao B. et al., 2023; Song et al., 2024). It is worth noting that most of these models are based on retrospective analyses, hence we still need prospective studies to validate their clinical application.

#### 3.3.4 Role of RNA methylation in GC: summary

**FIGURE 3:**
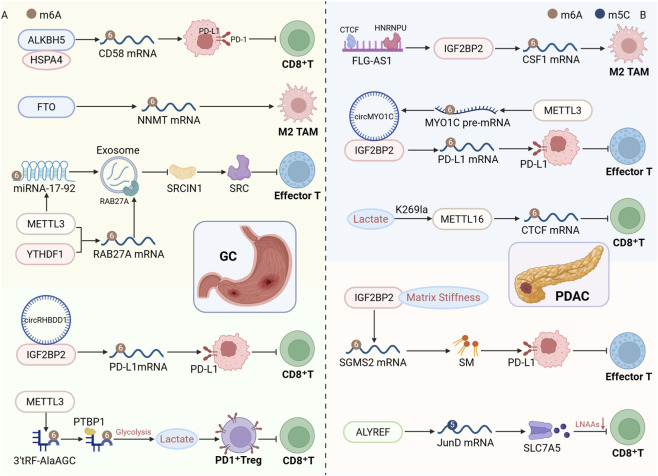
Regulatory mechanism of RNA methylation in the immune microenvironment of GC and PDAC. **(A)** In GC, m6A inhibits T cell function through the ALKBH5-CD58 axis and the METTL3-exosome signaling axis and drives macrophage M2 polarization via the FTO-NNMT pathway. Furthermore, m6A upregulates PD-L1 expression through IGF2BP2, promoting immune evasion; it also activates PD-1 signaling in Treg via the glycolysis-lactate axis, enhancing immunosuppression. **(B)** In PDAC, IGF2BP2 drives macrophage M2 polarization and inhibits T cell function; lactate-modified METTL16 further impairs T cell activity; mechanical signals from the matrix stabilize the IGF2BP2-SGMS2-SM axis, promoting PD-L1 lipid raft localization to enhance immune evasion. Furthermore, the m5C reader ALYREF inhibits CD8^+^ T cell function by reprogramming amino acid metabolism.

In summary, the RNA methylation network in GC drives tumor progression and peritoneal dissemination through three hallmark regulatory axes (Figure 3A). First, the “exosome-macrophage-peritoneum” communication axis facilitates exosome-mediated macrophage reprogramming, acting as a primary driver of peritoneal seeding. Second, the “stroma-immune” axis underscores the role of CAFs in leveraging m6A modifications to induce M2 macrophage polarization. Finally, the “epigenetic-exhaustion” axis, in synergy with lactate-Treg metabolic signaling, activates the PD-1/PD-L1 pathway to shape the “immune desert” landscape characteristic of CIN/GS molecular subtypes. These unique regulatory networks provide precise molecular targets for reversing immune suppression and inhibiting distant metastasis in GC.

### 3.4 RNA methylation and PDAC

#### 3.4.1 Role of m6A in immune regulation of PDAC

PDAC is one of the most aggressive tumors, with only about a 13% chance of 5-year survival (Jiang S. et al., 2025). The greatest challenge in treating this disease is its unique and highly immunosuppressive TIME, which shows classic signs of a “cold tumor.” These include a dense fibrotic stromal barrier, an imbalance in immune cell composition, and altered local metabolic conditions (Enzler and Frankel, 2025; Li F. et al., 2024). All these factors act together to create an immunosuppressive ecosystem. Currently, researchers are focusing on finding key molecular targets that can effectively reshape this immunosuppressive microenvironment. In recent years, RNA methylation modifications, such as m6A, have opened up new ideas and strategies for PDAC immunotherapy research.

##### 3.4.1.1 M6A modulates immune cells

The TIME of PDAC exhibits the typical characteristics of both an immune desert and immune suppression, not only marked by a severe deficiency of effector T cells but also by an abnormal expansion of immune-suppressive populations represented by M2 macrophages. Recent research indicates that the m6A modification is crucial for creating this imbalance. The CCCTC-binding factor (CTCF) forms a complex with lncRNA FLG-AS1 and HNRNPU. This complex activates the m6A reader IGF2BP2 to stabilize the mRNA for MYC and colony-stimulating factor 1 (CSF1), which promotes tumor growth. Additionally, it directly regulates the splicing and secretion of CSF1, thereby inducing M2 macrophage polarization through the CSF1-CSF1R axis (Liu et al., 2026). Therefore, targeting the key nodes in this network (such as IGF2BP2) could be a promising strategy to enhance immunotherapy effectiveness in PDAC.

##### 3.4.1.2 M6A regulates immune evasion

The m6A modification is crucial in the immune evasion process of PDAC by regulating immune checkpoints and affecting key aspects such as tumor antigen presentation. In regulating immune checkpoints, METTL3 promotes the circularization of circMYO1C, which helps stabilize PD-L1 mRNA and significantly enhances its expression level (Guan et al., 2023). In antigen presentation, METTL3-mediated m6A modification activates the Type I interferon signaling pathway by stabilizing endogenous dsRNA, thereby upregulating MHC-I expression and enhancing antigen presentation, ultimately strengthening the anti-tumor immune response (Zhu et al., 2023). Future treatments should target the m6A network (such as the METTL3/IGF2BP2 axis) to overcome immune resistance in PDAC.

##### 3.4.1.3 M6A reprograms tumor metabolism and fortifies stroma

The m6A modification not only directly regulates immune and tumor cell functions but also indirectly shapes the immunosuppressive environment of PDAC by reprogramming tumor metabolic characteristics and responding to the physical microenvironment. In a diabetes-associated PDAC model, lactate can be transported into tumor-associated Schwann cells (TASC) through MCT1/4, inducing lactylation at the K269 site of the METTL16 protein. This modification enhances the ability of METTL16 to stabilize CTCF mRNA in an m6A-dependent way, which then activates immunosuppressive ligands, ultimately impairing CD8^+^ T cell function and resulting in resistance to PD-1 therapy (Liu et al., 2025c). This study reveals a new way in which metabolic products regulate the immune microenvironment of PDAC through epitranscriptomic mechanisms. In addition to metabolic signals, physical factors also play a key role in the formation of the immunosuppressive microenvironment in PDAC. Studies show that stromal stiffness in PDAC can stabilize the m6A reader IGF2BP2, upregulating the expression of sphingomyelin synthase 2 (SGMS2) and increasing the synthesis of sphingomyelin (SM), and thereby promotes the specific localization of PD-L1 on lipid rafts of cell membranes, significantly boosting ability of tumor cells to evade the immune system (Tang et al., 2025). This finding highlights the important role of the physical microenvironment in regulating tumor immune behavior.

#### 3.4.2 Role of other RNA methylations in immune regulation of PDAC

Compared to m6A, research on the role of other types of RNA methylation modifications in the immune regulation of PDAC is relatively limited, and the relevant mechanisms are still not fully understood. Nonetheless, some early evidence suggests that they could play a significant role. For example, the m5C reader ALYREF facilitates the upregulation of SLC7A5 expression by recognizing and stabilizing JunD mRNA. This process activates the mTORC1 signaling pathway, which promotes tumor proliferation and depletes large neutral amino acids (LNAAs) in the TME, leading to impaired CD8^+^ T cell function, which ultimately creates an immunosuppressive microenvironment (Meng et al., 2024). This finding not only suggests that the m5C modification helps cancer evade the immune system by regulating amino acid metabolism but also offers valuable insights for investigating the roles of other RNA modifications in the immune regulatory network of PDAC. Given the unique challenges posed by the dense stroma and metabolic complexity of PDAC, systematic studies are warranted to deconstruct the intricate crosstalk between these factors in driving immune evasion.

#### 3.4.3 RNA methylation-based immune prognostic models for PDAC

In recent years, immune prognostic models based on RNA methylation characteristics have emerged as a cutting-edge focus in PDAC research. Multiple studies have shown that a low m6A score is associated with favorable prognosis, enhanced immune infiltration, and better immunotherapy responses (Zhou et al., 2021; Guo Y. et al., 2021; Xu et al., 2022). Moreover, a combined risk model that includes m6A, m5C and m1A can effectively distinguish subgroups with different immune characteristics and treatment sensitivities (Huang et al., 2023). Although these models show great promise in predicting outcomes, their development relies heavily on bioinformatics analyses. However, their clinical value still needs rigorous validation, requiring in-depth mechanistic experiments and large-scale prospective clinical trials.

#### 3.4.4 Role of RNA methylation in PDAC: summary

In summary, the RNA methylation network in PDAC establishes a stable “cold tumor” ecosystem by integrating biomechanical cues and metabolic stress (Figure 3B). First, the “stromal stiffness-epigenetic” axis illustrates how the characteristic physical barrier of PDAC drives immune evasion through m6A modification. Second, the “metabolic-epigenetic” axis involves lactate-induced METTL16 lactylation and m5C-mediated amino acid depletion, which collectively transform the metabolic stress of PDAC into T cell dysfunction. Together, these specific axes define the distinctive “fibrotic stroma–epigenetic” landscape of PDAC, providing precise targets for reversing its profound immune tolerance.

### 3.5 RNA methylation and ESCA

#### 3.5.1 Role of RNA methylation in immune regulation of ESCA

ESCA is one of the most aggressive and lethal malignancies in the world (Zheng et al., 2020). Despite continuous advancements in traditional therapies, the survival rate for advanced ESCA patients remains poor. While ICIs have shown efficacy in a subset of patients, effective treatment remains limited. The main issue is the high complexity and heterogeneity of the TIME (He et al., 2021). However, compared to other gastrointestinal tumors such as HCC, CRC and GC, there is still not much experimental evidence about RNA modifications and how they regulate the immune microenvironment in ESCA. Currently, most studies focus on the effects of m6A modification on typical cancer traits such as tumor cell proliferation, migration and invasion (Zhao et al., 2024; He et al., 2023), while its role in the immune microenvironment of ESCA remains largely unknown.

Despite the limited research in this field, several key findings in recent years have revealed the complex network regulating the immune microenvironment of ESCA via m6A modification. In the context of TAM reprogramming, m6A regulatory factors exhibit significant bidirectional modulation: on the one hand, forkhead box F2 (FOXF2) mediates the ubiquitination and degradation of FTO by activating RING finger protein 144A (RNF144A), thereby suppressing tumor proliferation and inhibiting M2 polarization of TAMs, exerting an anti-tumor effect (Han T. et al., 2024); on the other hand, ZC3H13 stabilizes CXCL8 mRNA via an m6A-dependent mechanism, activating the CXCL8-CXCR2 axis and directly driving the M2 macrophage polarization (Yan et al., 2025). This study suggests that targeting this axis could help reverse the immunosuppressive microenvironment of ESCA. In T cell-mediated immune surveillance, intrinsic host regulatory mechanisms coexist with interference from exogenous pathogens. Specifically, RBM15 upregulates procollagen-lysine, two-oxoglutarate 5-dioxygenase 3 (PLOD3) expression in an m6A-dependent manner, enhancing CD4^+^ T cell infiltration and correlating with improved prognosis (Lin et al., 2024). Conversely, infection with *Porphyromonas gingivalis* (*Pg*), a pathogen closely associated with ESCA, exploits the YTHDF2-mediated Fas cell surface death receptor/Fas ligand (Fas/FasL) degradation pathway to impair T cell cytotoxicity, thereby facilitating immune evasion (Yang et al., 2024). In terms of metabolic reprogramming, serine hydroxymethyltransferase 2 (SHMT2) boosts SAM levels through one-carbon metabolism, stabilizing c-myc mRNA in a METTL3/FTO/ALKBH5/IGF2BP2-dependent manner, which drives tumor progression and promotes immune evasion (Qiao et al., 2023). This indicates that intervening in this metabolic pathway could synergistically enhance immunotherapeutic efficacy.

Although ESCA is highly invasive and lethal, research on its RNA methylation profile remains significantly limited compared to other gastrointestinal tumors such as HCC and CRC. First, there is a marked “modification bias”: current research mainly focuses on m6A, while the roles of modifications such as m5C, m1A, and m7G in immune regulation remain largely unexplored. Second, there is a “mechanistic gap” in the regulation of the TIME: existing studies mainly concentrate on tumor-intrinsic phenotypes (such as proliferation and metastasis), while the mechanisms by which RNA modifications systematically regulate immune cell infiltration, antigen presentation, and immune checkpoint expression have not been fully elucidated. Third, there is a lack of etiology-specific research: although the occurrence and development of ESCA are profoundly influenced by the esophageal microbiota and mechanical stress, how RNA modifications mediate the integration of these exogenous factors to reshape the immune microenvironment remains unclear. Systematically bridging these gaps is essential for the development of precise epigenetic immunotherapies for ESCA.

#### 3.5.2 RNA methylation-based immune prognostic models for ESCA

An immune prognostic model constructed based on m6A-related features can open up new frontiers for the precise diagnosis and treatment of ESCA. These models not only effectively predict how patients are stratified by survival but they are also closely tied to the characteristics of tumor immune infiltration, offering new insights for assessing prognosis and personalizing immunotherapy (Guo W. et al., 2021; Wang J. et al., 2024; Nie et al., 2023). However, the clinical translational value of these models still needs to be validated, and future efforts should focus on in-depth studies of the mechanisms and validating these models in large clinical cohorts to facilitate the transition of theoretical models into practical clinical tools.

#### 3.5.3 Summary of ESCA

In summary, the unique “pathogen-epigenetic-immune” axis of ESCA establishes a critical link between disease-specific etiology and immune evasion. However, significant knowledge gaps remain concerning the diversity of RNA modifications, particularly non-m6A marks. Targeting these pathogen-driven extrinsic vulnerabilities, alongside a deeper exploration of the broader epitranscriptomic landscape, will be pivotal for overcoming immune resistance and advancing precision targeted therapies in ESCA.

## 4 Conclusion

This review systematically elucidates the key regulatory functions of RNA methylation modifications such as m6A, m5C, m1A, and m7G in the immune microenvironment of gastrointestinal tumors. These modifications contribute to the formation of an immunosuppressive microenvironment by targeting various cellular components and signaling pathways. Importantly, RNA modification plays a role in the TIME that is not limited to immunosuppression. Using METTL3 in PDAC as an example (Zhu et al., 2023), this factor can enhance anti-tumor immunity driven by Type I interferon, demonstrating the context-dependent dual functions that RNA modification may possess. This “double-edged sword” effect adds complexity to therapeutic interventions, indicating that a detailed understanding of the specific targets and signaling pathways involved in RNA methylation across different immune cell subsets is required before translating these findings into clinical practice.

However, it should be noted that the prominent position of m6A in the current literature does not necessarily imply that it has intrinsic biological advantages or is more universal in the immune response of gastrointestinal tumors. On the contrary, this asymmetry mainly reflects the advanced level of research tools, detection techniques, and functional characterization used for m6A. Other modification types, such as m5C, m1A, and m7G, although studied relatively less, have shown significant regulatory potential and may prove to be equally important as research progresses. Whether m6A is always superior to other modifications at the biological level, or whether their roles are underestimated in current research but still hold equal importance in the future, remains an open question to be explored in the future.

In addition to the individual effects of each RNA modification, emerging evidence suggests that intricate cross-talk and synergistic effects exist among diverse epitranscriptomic marks to orchestrate the TIME. For example, the EME scoring system, which integrates m5C and m6A markers, not only outperforms single-modification models in predicting patient prognosis but also accurately identifies high-risk populations for immune evasion, providing important evidence to inform precise immunotherapy strategies for HCC (Tian et al., 2023). The superiority of this integrative model is likely due to the complex molecular interactions between different modification types. However, despite these potential mechanisms, there is still a lack of systematic research on how different modifications regulate each other—whether they jointly regulate targets, compete for binding sites, or function in parallel but complementary pathways. Therefore, an in-depth investigation of the interaction network among m6A, m5C, m1A, and m7G is crucial to comprehensively understand the regulatory complexity of the TIME and to develop multi-target immunotherapy strategies.

Looking ahead, with the development of technologies such as single-cell epitranscriptomics and spatial transcriptomics, we can obtain a clearer picture of how RNA methylation regulates TME cell interactions over time and space. In addition to these high-resolution technologies, more attention should be paid to the practical clinical transformation. For example, developing non-invasive liquid biopsy techniques by identifying specific RNA methylation patterns in circulating cell-free RNA (cfRNA) or exosomes may provide a powerful tool for early diagnosis and real-time monitoring of immunotherapy efficacy (Nesselbush et al., 2025; Ma et al., 2024). Furthermore, exploring combination therapy strategies, such as using RNA methyltransferase inhibitors to sensitize “cold” tumors to ICIs, represents a promising direction to overcome current immunotherapy resistance (Liu et al., 2023). The therapeutic potential of this strategy has been validated in preclinical models using modulators such as the METTL3 inhibitor STM2457 (Yankova et al., 2021) and the FTO inhibitor Dac51 (Liu et al., 2021), both of which have demonstrated the ability to reshape the TIME. Currently, the field has advanced into the clinical translation phase, highlighted by the first-in-class METTL3 inhibitor STC-15 undergoing Phase I clinical trials (NCT05584111). However, the clinical translation of these agents still faces bottlenecks such as target specificity, systemic toxicity, and complex bidirectional immunomodulatory effects. Therefore, future efforts should focus on developing tumor-specific delivery systems (such as nanoparticles) and identifying predictive biomarkers to precisely guide clinical treatment.
